# Hypereosinophilic Syndrome Endocarditis With Severe Biventricular Failure Complicated by Sepsis: A Challenging Case

**DOI:** 10.7759/cureus.44963

**Published:** 2023-09-09

**Authors:** Zahid Khan, Umesh Kumar Pabani

**Affiliations:** 1 Acute Medicine, Mid and South Essex NHS Foundation Trust, Southend-on-Sea, GBR; 2 Cardiology, Barts Heart Centre, London, GBR; 3 Cardiology and General Medicine, Barking, Havering and Redbridge University Hospitals NHS Trust, London, GBR; 4 Cardiology, Royal Free Hospital, London, GBR; 5 Internal Medicine, Mid and South Essex NHS Foundation Trust, Southend-on-Sea, GBR

**Keywords:** copd (chronic obstructive pulmonary disease), loeffler endocarditis, septic shock in endocarditis, prednisolone and eosinophilia, inotropes, eosinophilic myocarditis, nosocomial aspiration pneumonia, eosinophilic endocarditis, idiopathic hypereosinophilic syndrome, hypereosinophilic syndromes

## Abstract

Hypereosinophilic syndrome (HES) is a myeloproliferative disorder characterized by persistent hypereosinophilia that is associated with multi-organ damage. Eosinophilic endocarditis is a serious complication of HES. The exact prevalence of the disease is unknown, and it is characterized by a persistently elevated eosinophil count, resulting in multi-organ involvement due to eosinophilic infiltration.

We present a case of a 65-year-old Caucasian male patient who presented with one-week symptoms of feeling unwell and intermittent pleuritic chest pain. His medical history was significant for the idiopathic hypereosinophilic syndrome, eosinophilic myocarditis, hypertension (HTN), type 2 diabetes mellitus (T2DM), and chronic obstructive pulmonary disease (COPD). Inflammatory markers were raised, including eosinophil count, and a transthoracic echocardiogram (TTE) showed a mass attached to the mitral valve (MV) leaflets, suggesting vegetation or thrombus. The patient was commenced on intravenous antibiotics, inotropes for septic shock, and low molecular weight heparin (LMWH) for a possible thrombus. He showed mild biochemical improvement initially without any clinical improvement before further deterioration secondary to aspiration pneumonia. He was seen by the palliative care team and mental health team for confusion and agitation and was put on the palliative care pathway. All active medical treatment was stopped, and the patient succumbed to his illness three weeks into his admission.

## Introduction

Loeffler endocarditis (LE) is a rare, life-threatening heart condition caused by eosinophilic infiltration resulting in multi-organ dysfunction [1]. Cardiac involvement occurs in about 50%-60% of cases and is characterized by unexplained persistent eosinophilia with more than 1500 cells/μL for at least six months and is associated with organ dysfunctions secondary to eosinophilic infiltration [1,2]. The first case of Loeffler endocarditis secondary to eosinophil infiltration was described by Loeffler in 1936 [1]. The cardiac involvement in hypereosinophilic syndrome (HES) Loeffler endocarditis involves myocardium and endocardial damage due to eosinophil infiltration and degranulation, resulting in tissue inflammation and fibrosis [1]. Loeffler endocarditis, when associated with intracardiac thrombus, carries a high mortality rate, and the prognosis is very poor [3]. Cardiac involvement in Loeffler syndrome results in endocardial thickening, atrial dilatation, and a restrictive pattern from eosinophil degranulation, resulting in diastolic dysfunction on echocardiography. These findings are occasionally accompanied by echogenic fibrosis or thrombosis, frequently located in the apical region of the left and right ventricles, resulting in ventricular obliteration [1,4].

The data regarding Loeffler syndrome and its prognosis are scarce [3]. Early diagnosis and management are important in management, and there is no consensus or set of guidelines available for managing Loeffler syndrome [4]. We present a case of Loeffler syndrome in a 65-year-old patient who presented with septic shock and was treated with antibiotics and low molecular weight heparin (LMWH) but unfortunately succumbed to the disease.

## Case presentation

We present a case of a 65-year-old Caucasian patient who presented to a district general hospital with one week of symptoms of feeling unwell and having chest pain. On arrival, he was vitally stable, apart from mild tachypnoea and tachycardia. His medical history was significant for idiopathic hypereosinophilic syndrome diagnosed in 2014, eosinophilic myocarditis in 2022, and he had undergone cardiac magnetic resonance (CMR) imaging that demonstrated a dilated left ventricle with a left ventricular ejection fraction (LVEF) of 20%, a sub-endocardial scar in the septum and inferior walls on late gadolinium enhancement, chronic obstructive pulmonary disease (COPD), type 2 diabetes mellitus (T2DM), a cerebrovascular accident (CVA) in 2022, hypertension, and a previous episode of acute inflammation of unknown cause in 1998 with a raised troponin T and eosinophil count of four. A repeat echocardiogram earlier this year demonstrated recovery of his LVEF to 45% with heart failure therapy. Regular medications included prednisolone 5 mg once daily (OD), pregabalin 150 mg twice daily (BD), inhalers, cholecalciferol 1000 international units (IU) once daily, ferrous sulfate 200 mg OD, famotidine 20 mg OD, folic acid 5 mg OD, and sertraline 100 mg OD.

He was admitted to the cardiology unit, developed type 2 respiratory failure, and was commenced on non-invasive ventilation (NIV). He became hypotensive, developing cardiogenic/septic shock and acute kidney injury (AKI) overnight, requiring intensive care unit (ICU) admission at the district general hospital (DGH). He became anuric, hypotensive, and developed metabolic acidosis, requiring intravenous fluids and the inotropes dobutamine 250 mg infusion and nor-adrenaline at 4.44 ml/hour and 0.2 mcg/kg/min, respectively. The nor-adrenaline was weaned off to 0.1 mcg/kg/min later. His blood pressure and urine output improved following inotropes and intravenous fluids. He was commenced on empirical antibiotic therapy, including amoxicillin 2 gm every four hours, flucloxacillin 2 gm every six hours, and gentamycin 200 mg once daily, following discussion with microbiology and a multidisciplinary team meeting (MDT). He was started on low-molecular-weight heparin (enoxaparin 100 mg) twice daily for a possible mitral valve thrombus, as the mass was suspected to be either a thrombus or vegetation.

The echocardiogram the next day showed possible mitral valve infective endocarditis, and the patient had an urgent ICU-ICU transfer to our hospital. On arrival, vitals were as follows: blood pressure (BP) of 115/63 mmHg on dobutamine and non-adrenaline infusions, mean arterial pressure of 76 mmHg, a heart rate of 109 bpm, respiratory rate (RR) of 22 breaths per minute, SpO_2_ of 98% on room air, and his temperature was 36.8°C. On clinical examination, he had reduced air entry to the right lung base, and his heart sounds were normal. He was fully alert and obeying commands. He had a standard arterial blood gas (ABG) despite requiring the inotropes dobutamine at 4.44 ml/hour and noradrenaline at 0.2 mcg/kg. The echocardiogram showed mitral valve mass suggestive of infective endocarditis and LVEF 10%-20% (Videos 1-4).

**Video 1:** Four-chamber view of the patient's echocardiogram

**Video 2:** Four-chamber view of the patient's echocardiogram showing severely impaired left ventricular function and mitral valve mass

**Video 3:** Parasternal long-axis view showing mitral regurgitation

**Video 4:** Apical four-chamber view

His blood tests showed a white cell count of 15.0 x 10^9/L, a neutrophil count of 12.7 x 10^9/L, a C-reactive protein (CRP) of 34 mg/L, troponin T of 1672 ng/L, an eosinophil count of 2 x 10^9/L, and procalcitonin of 0.4 ng/mL. Other blood tests showed creatinine of 182 umol/L, sodium of 127 mmol/L, and potassium of 5.0 mmol/L (Table 1).

**Table 1:** The patient's laboratory test results during admission

Lab Test	Day 1	Day 5	Day 10	Day 15	Reference Values
Haemoglobin	107	97	79	104	120-150 g/L
White Cell Count	14.9	8.0	12.2	16.4	4-10 x10^9/L
Platelets	195	167	185	197	150-410 x10^9/L
Neutrophil	13.1	5.8	14.4	15.4	2-7 x10^9/L
Eosinophil Count	2.0	1.4	0.2	0.1	0-0.2 x10^9/L
Urea	5.6	5.7	1.	4.1	2.5-7.8 mmol/L
Creatinine	143	169	161	205	45-84 umol/L
Sodium	126	126	128	131	133-146 mmol/L
Potassium	4.3	4.1	3.8	4.4	3.5-5.3 mmol/L
Alanine Aminotransferase	112	94	78	45	0-33 unit/L
Alkaline Phosphatase	92	85	81	82	30-130 unit/L
Bilirubin	14	18	17	21	0-21 umol/L
C-Reactive Protein	51	83	117	141	0-5 mg/L
Adjusted Calcium	2.36	2.35	2.32	2.37	2.2-2.6 mmol/L
Phosphate	1.16	1.27	0.87	0.29	0.8-1.5 mmol/L
Magnesium	0.9	1.0	0.8	0.8	0.7-1 mmol/L
Thyroid Stimulating Hormone	2.90	-	-	-	0.27-4.2 mU/L
Thyroxine (T4) Thyroid Hormone	16.9	-	-	-	10.5-24.5 pmol/L
Troponin T	1672	1172	-	-	0-14 ng/L

Blood cultures grew *Candida albicans* and coagulase-negative *Staphylococcus haemolyticus*, which could be contaminants. The ABG on admission showed an acidity (pH) of 7.43 kilopascals (kPa), a partial pressure of carbon dioxide (pCO2) of 4.63 kPa, a partial pressure of oxygen (PaO2) of 9.9 kPa, lactate of 1.5 mmol/L, bicarbonate of 23.3 mmol/L, and a base excess of -0.6 mmol/L.

**Figure 1:**
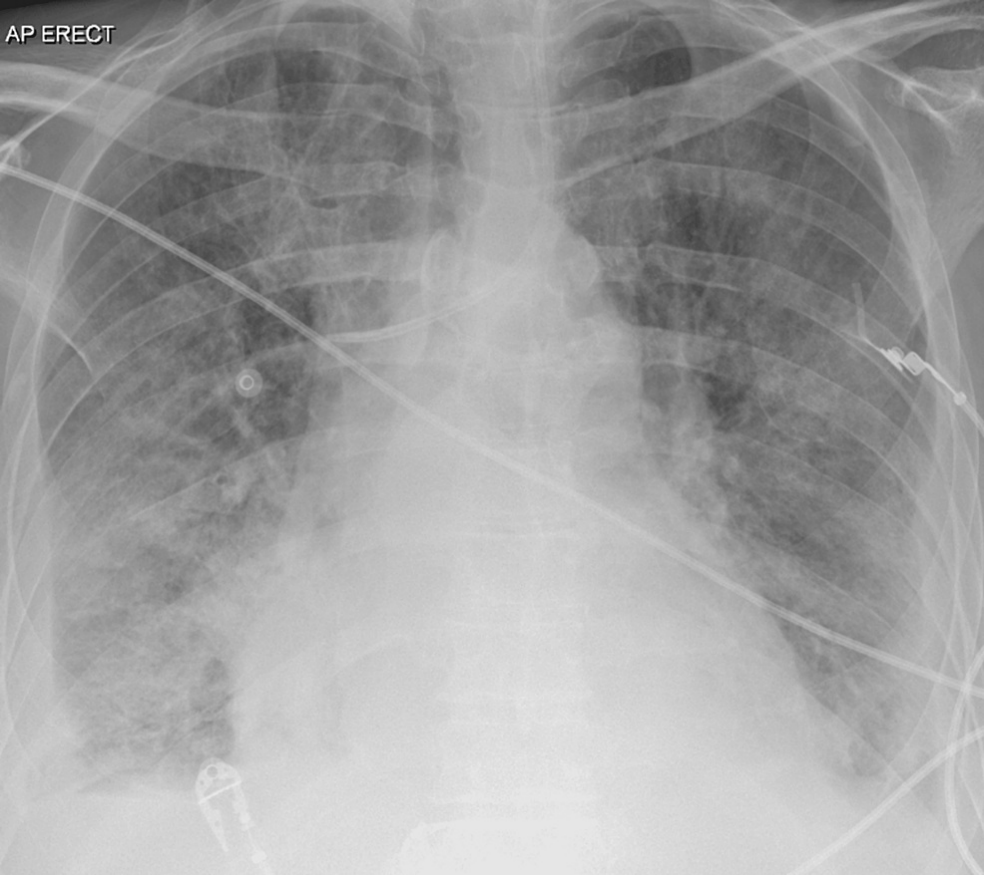
Chest radiography two weeks later shows pulmonary congestion +/- consolidation.

The inflammatory markers and renal functions showed initial improvement during the course of the stay, but there was no clinical improvement in the patient's condition. An echocardiogram two weeks later showed persistent mitral valve (MV) vegetation and chest radiography showed worsening pulmonary congestion +/- consolidation (Videos 5-6, Figure 1). 

**Video 5:** A parasternal long-axis view of the echocardiogram two weeks later shows persistent mitral valve vegetation.

**Video 6:** The apical four-chamber view shows mitral regurgitation and persistent mitral valve vegetation.

He was started on intravenous furosemide (20 mg twice daily), and spironolactone was increased to 25 mg twice daily. A peripherally inserted central catheter (PICC) was inserted to facilitate ongoing management. The pulmonary congestion and ongoing sepsis made management extremely challenging, and metolazone 2.5 mg on alternate days was added to achieve optimal fluid balance. He was commenced on bumetanide 2 mg twice daily four days later, and his oxygen requirement was reduced, and intravenous furosemide and metolazone were stopped. His inflammatory markers continued to rise, and his oxygen requirement increased the following morning; the patient was diagnosed with aspiration pneumonia. Metronidazole 500 mg three times daily, temocillin 1 gram was added to his antibiotic regime for aspiration pneumonia and gentamycin was stopped. His white cell count, neutrophil, and CRP upward trend eosinophil count were reduced to 0.1, and the patient showed rapid clinical deterioration. The patient was discussed with the palliative care team and mental health team, given his further deterioration despite being on antibiotics for about three weeks. Renal functions showed deterioration along with rising inflammatory markers, and the patient was initially on 2 mg of melatonin, which was then increased to 4 mg once at night to help with his sleeping. He was becoming more agitated and confused, and based on a discussion with family and the patient's initial wishes, the patient was commenced on the end-of-life pathway, and active treatment was stopped. The patient, unfortunately, succumbed to the illness after three weeks of admission.

## Discussion

Hypereosinophilic syndrome (HES) is an uncommon type of myeloproliferative disorder with a prevalence of 0.15 cases per 100,000 persons, and cardiac involvement is seen in 50%-60% of cases [1,5]. Currently, the available evidence is limited, and reported cases suggest that it is a life-threatening condition with high mortality. Hypereosinophilic syndrome can essentially involve any organ of the body; however, the heart is the most frequently affected viscera. Cardiac involvement typically advances through stages of eosinophil infiltration, acute damage to cardiac tissue causing thrombosis, and endomyocardial fibrosis resulting in diastolic dysfunction. This constellation of cardiac involvement is termed "Loeffler's endocarditis" [6]. Patients are suspected to have hypereosinophilia when their eosinophil count is ≥1500/mm3 in the peripheral blood and this may be due to parasitic infections, reactive, neoplastic, or idiopathic causes [7]. The diagnostic criteria for HES include an increased eosinophil count (>1500/mm3) on two separate tests (≥1 month); tissue hypereosinophilia with bone marrow examination showing >20% eosinophils or significant eosinophilic granule deposition in tissues leading to organ damage; and the presentation can vary depending on the organ involved [7].

The majority of the patients with Loeffler’s endocarditis present with signs and symptoms of acute heart failure, including shortness of breath, bipedal oedema, chest pain, fatigue, and reduced exercise tolerance. A minority could also present with signs of pericarditis. Most commonly, HES affects the mitral valve, leading to mitral valve dysfunction and resulting heart failure [6,8,9]. This case report highlights the nine years of the natural history of this disease and its cardiac complications, despite the patient being on treatment. Our patient was diagnosed with hypereosinophilic syndrome in 2014, and then in 2022, he developed eosinophilic myocarditis despite being on a maintenance dose of prednisolone. In 2023, he presented with chest pain and cardiogenic shock, and an echocardiogram confirmed evidence of mitral valve endocarditis. Unfortunately, this admission proved to be his ultimate presentation with complications of his condition.

An echocardiogram is the baseline investigation to assess for cardiac function and valvular dysfunction; however, a cardiac magnetic resonance imaging scan (CMR) is the gold standard non-invasive investigation for the diagnosis, monitoring, and prognostic stratification of patients with HES [7,9]. Cardiac manifestations occur in about half of HES patients and carry high morbidity and mortality [7]. A CMR shows the characteristic pattern of fibrosis in myocardial tissue on subendocardial late gadolinium enhancement, reducing the need for invasive biopsy in HES patients. If the endocardial fibrosis is complicated by a left ventricular apical thrombus, then CMR would illustrate it as a non-enhancing hypointensity between the enhancing endocardium and bright blood pool with a sandwich-like appearance, as correctly demonstrated [9].

Treatment of Loeffler's endocarditis typically comprises symptomatic management if the patient is in heart failure, affected valve replacement, and long-term immunosuppression to alter the disease course. Symptomatic management comprises diuretics, beta-blockers, angiotensin-converting enzyme (ACE) inhibitors, and aldosterone antagonists, and if the patient is in cardiogenic shock, then it is essential to manage the patient in intensive care with inotropes and vasopressor support. Valve replacement is an important aspect of management if the disease process presents as a vegetative or thrombotic mass on valves. Bioprosthetic valves should take precedence over mechanical valves due to the increased risk of valve thrombosis despite anticoagulation. Steroids are the first-line immunosuppressive treatment for HES. Depending on the aetiology, hydroxyurea can also be considered to reduce the eosinophil count [9,10,11]. 

Genetic testing has changed the course of HES management. Patients identified with the FIP1L1-PDGFRA mutation on genetic testing should be considered for management with tyrosine kinase inhibitors such as imatinib. A low dose of imatinib mesylate (100 milligrams per day) is sufficient to obtain haematological and molecular remission [9]. Patients with HES are also at increased risk of ischemic strokes, especially in watershed areas. Tennenbaum J et al., in their study, recommended that patients with ischemic strokes of undetermined aetiology be screened for HES and FIP1L1-PDGRA mutations, as treatment with imatinib will reduce the risk of future strokes [12].

## Conclusions

Hypereosinophilic syndrome is an uncommon condition that can present with myocarditis and endocarditis. The condition carries a very high mortality rate, and early diagnosis and management are key to improving the chances of survival. Our patient presented with cardiogenic shock and, despite maximum therapy, did not show any improvement. These patients usually benefit from steroids and heart failure therapy. Patients in cardiogenic shock require inotropic support, and HES endocarditis patients with thrombosis should also be treated with anticoagulation therapy.
